# A195 DURABILITY OF SEROLOGICAL RESPONSES AFTER SECOND, THIRD AND FOURTH DOSE OF SARS-COV-2 VACCINATION IN INFLAMMATORY BOWEL DISEASE: A PROSPECTIVE COHORT STUDY

**DOI:** 10.1093/jcag/gwac036.195

**Published:** 2023-03-07

**Authors:** N Sharifi, C Ma, C Seow, J Quan, L Hracs, L Caplan, A Markovinović, M Herauf, J Windsor, S Coward, M Buie, J Gorospe, R Panaccione, G Kaplan

**Affiliations:** Department of Medicine, University of Calgary, Calgary, Canada

## Abstract

**Background:**

Adequate serological responses following two-dose regimens and additional doses of SARS-CoV-2 vaccination have been demonstrated for the vast majority of those with IBD. However, antibody levels following 2^nd^, 3^rd,^ and 4^th^ dose SARS-CoV-2 vaccination may decrease over time in the IBD population.

**Purpose:**

We assessed the durability of serological responses to 2^nd^, 3^rd^, and 4^th^ dose SARS-CoV-2 vaccination over time in a cohort of IBD patients.

**Method:**

Adults with IBD who received at least one dose of a SARS-CoV-2 vaccine (*n*=559) were evaluated for serological response to the spike protein of SARS-CoV-2 using the Abbott IgG II Quant assay with a seroconversion threshold of ≥ 50 AU/mL. The geometric mean titer (GMT) with 95% confidence intervals (CI) were calculated and stratified by weeks (1–8, 8–16, 16–24, 24+ weeks) after each vaccine dose. We compared stratified GMTs with Mann–Whitney U tests using a significance level of 0.05.

**Result(s):**

Our cohort (*n*=559) comprised the following patient characteristics: 82.8% were 18–65 years-old (*n* = 463), 53.1% were female (*n* =297), and 71.6% had Crohn’s disease (*n* =400). IBD medications were classified in the following mutually exclusive groups: No immunosuppressives 10.5% (*n* = 59), anti-TNF monotherapy 35.8% (*n* = 200), immunomodulatory monotherapy 2.1% (*n* =12 ), vedolizumab 11.8% (*n* =66 ), ustekinumab 20.4% (*n* =114 ), tofacitinib 1.2% (*n* =7 ), combination therapy 15.9% (*n* = 89), and prednisone 2.1% (*n* =12). For vaccine type, 85.6% and 82.3% had Pfizer for 3^rd^ and 4^th^ dose, respectively, while the remainder had Moderna. Seroconversion rates 1–8 weeks after 3^rd^ and 4^th^ dose were both 99.9%.

Figure 1 compares GMTs with 95% CI by weeks after each vaccine dose. GMTs are highest 1–8 weeks after 2^nd^ dose (4053 AU/mL; 95% CI: 3468, 4737 AU/mL; *n*=337), 3^rd^ dose (12116 AU/mL; 10413, 14098 AU/mL; *n*=256), and 4^th^ dose (14337 AU/mL; 10429, 19710 AU/mL; *n*=67). Subsequently, antibody levels decay from 1–8 weeks to 8–16 weeks (*p*<0.001) for 2^nd^ dose (mean difference: –2224 AU/mL), 3^rd^ dose (mean difference: –7526 AU/mL), and 4^th^ dose (mean difference: –9715 AU/mL). Compared to 16–24 weeks after 2^nd^ dose, antibody levels 24+ weeks after were similar (GMTs: 795 AU/mL vs. 1043 AU/mL, *p*=0.52). For third dose, antibody levels 8–16 weeks and 16–24 weeks after vaccination were similar (4590 AU/mL vs. 4073 AU/mL, *p*=0.73) along with 16–24 weeks compared to 24+ weeks after vaccination (4073 AU/mL vs. 5876 AU/mL, *p*=0.18).

**Image:**

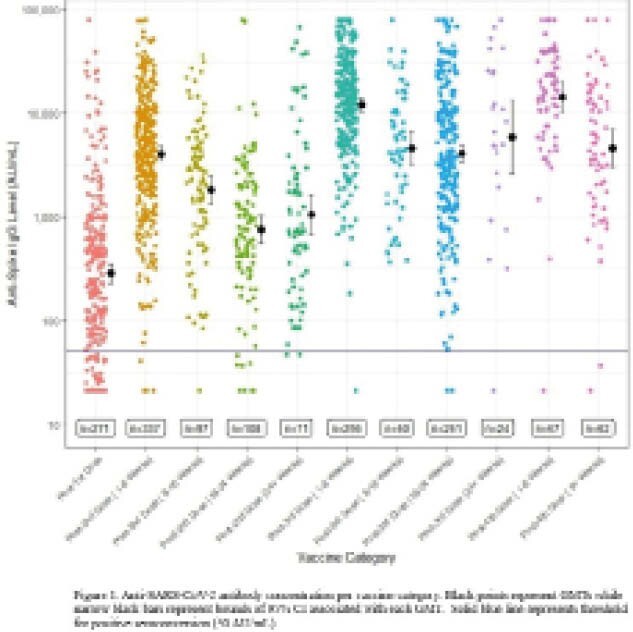

**Conclusion(s):**

Within 1–8 weeks after each dose of vaccine, serological responses spikes with each subsequent dose yielding a higher GMT. While antibody levels decay 8–16 weeks after each dose, similar GMT levels beyond 16 weeks may indicate durability of antibody levels over a longer duration of time.

**Disclosure of Interest:**

None Declared

